# A bibliometric analysis of cerebral palsy from 2003 to 2022

**DOI:** 10.3389/fneur.2024.1292587

**Published:** 2024-04-02

**Authors:** Yue Hu, Yadan Zheng, Yue Yang, Wenfeng Fang, Maomao Huang, Dan Li, Zhangyu Xu, Fangyuan Xu, Jianxiong Wang

**Affiliations:** ^1^Department of Rehabilitation Medicine, The Affiliated Hospital of Southwest Medical University, Luzhou, Sichuan, China; ^2^Department of Rehabilitation Medicine, Southwest Medical University, Luzhou, Sichuan, China; ^3^Rehabilitation Medicine and Engineering Key Laboratory of Luzhou, Luzhou, Sichuan, China

**Keywords:** cerebral palsy, bibliometric analysis, citation, CiteSpace, Bibliometrix

## Abstract

**Purpose:**

This bibliometric study explores cerebral palsy (CP) research from 2003 to 2022 to reveal the topic hotspots and collaborations.

**Methods:**

We retrieved studies on CP from the Web of Science Core Collection from 2003 to 2022 and then used CiteSpace and Bibliometrix to perform a bibliometric analysis and attain knowledge mapping, including publication outputs, funding, journals, authors, institutions, countries/territories, keywords, collaborative relationships, and topic hotspots.

**Results:**

In total, 8,223 articles were published from 2003 to 2022. During this period, the number of publications increased continuously. *Developmental Medicine and Child Neurology* was the most productive and frequently co-cited journal. Boyd was the most productive and influential author, with 143 publications and 4,011 citations. The United States and Vrije Universiteit Amsterdam were the most productive countries and institutions, respectively. Researchers and institutions from the USA, Australia, and Canada constituted the core research forces, with extensive collaborations worldwide. The most common keywords were gait (553), rehabilitation (440), spasticity (325), botulinum toxin (174), therapy (148), upper extremity (141), quality of life (140), disability (115), pain (98), electromyography (97), kinematics (90), balance (88), participation (85), and walking (79).

**Conclusion:**

This study provides a systematic and comprehensive analysis of the CP-related literature. It reveals that *Developmental Medicine and Child Neurology* is the most active journal in this field. The USA, Vrije Universiteit Amsterdam, and Boyd are the top countries, institutions, and authors, respectively. Emerging treatment methods, complication management, and functional recovery comprise the future research directions and potential topic hotspots for CP.

## 1 Introduction

Cerebral palsy (CP) is a nonprogressive brain injury syndrome that adversely affects the long-term health of patients. It is a central motor and postural developmental disorder caused by a variety of factors during the early brain development process, from before birth to 1 month after birth, and is often combined with sensory, cognitive, communication, and behavioral abnormalities. A systematic review in 2013 found that although the survival rates for high-risk preterm infants had improved, the overall prevalence rate of CP remained unchanged (1) and was as high as 2–3% (2, 3). Complications related to CP have also received widespread attention. A meta-analysis found a 93% (95% confidence interval [CI]: 71–99) prevalence rate of the equinus foot in patients with CP (4) and a 70% (95% CI: 62–78) overall average pain prevalence rate in adults with CP (5). This high epidemiology has resulted in a large cohort of patients with CP, presenting a serious public health problem and has a heavy global socioeconomic burden. CP is an interdisciplinary field with dedicated scholars in pediatrics, neurology, pediatric orthopedics, rehabilitation medicine, orthopedics, and genetics domains. The progress in related disciplines has also promoted CP research; for example, advances in genetics, imaging technologies, and the latest rehabilitation treatment technologies, including repetitive transcranial magnetic stimulation and exoskeleton robots. Specifically, the CP research progressed remarkably from 2003 to 2022, including the assessment of gross motor function, clinical surgical treatment modalities, and manipulative treatment strategies (6, 7). In addition to motor functions, other functions pertaining to individuals with CP have gradually attracted researchers' attention, such as pain, vision, quality of life, and outcomes. Therefore, it is vital to conduct qualitative and quantitative assessments of the literature to further promote the CP research and clarify future research directions.

Bibliometrics is used to evaluate and quantify the literature. The Web of Science Core Collection (WOSCC) is one of the most commonly used bibliometric analysis databases that provides basic information on annual output, authors, journals, institutions, countries, funds, and citations (8). CiteSpace is one of the most commonly used bibliometric analysis tools (9), while Bibliometrix, an R language program, is used for bibliometric analysis and visualization. Bibliometric studies have focused on certain aspects of CP, such as changing trends in disability and rehabilitation topics (10), trends in orthopedic surgical management of CP (11), spastic CP (12, 13), CP research in the pediatric field (14), CP imaging research (15), treatments for CP (16), and systematic reviews and meta-analyses of the CP intervention research (17). To the best of our knowledge, there has been no comprehensive bibliometric analysis of the CP-related research fields, especially citation and global collaboration analyses. Therefore, we conducted a bibliometric study and visual analysis of the CP literature from 2003 to 2022 to analyze the scientific research achievements, current status of cooperative relationships, research trends, and frontier changes in CP research.

## 2 Materials and methods

**Figure 1:**
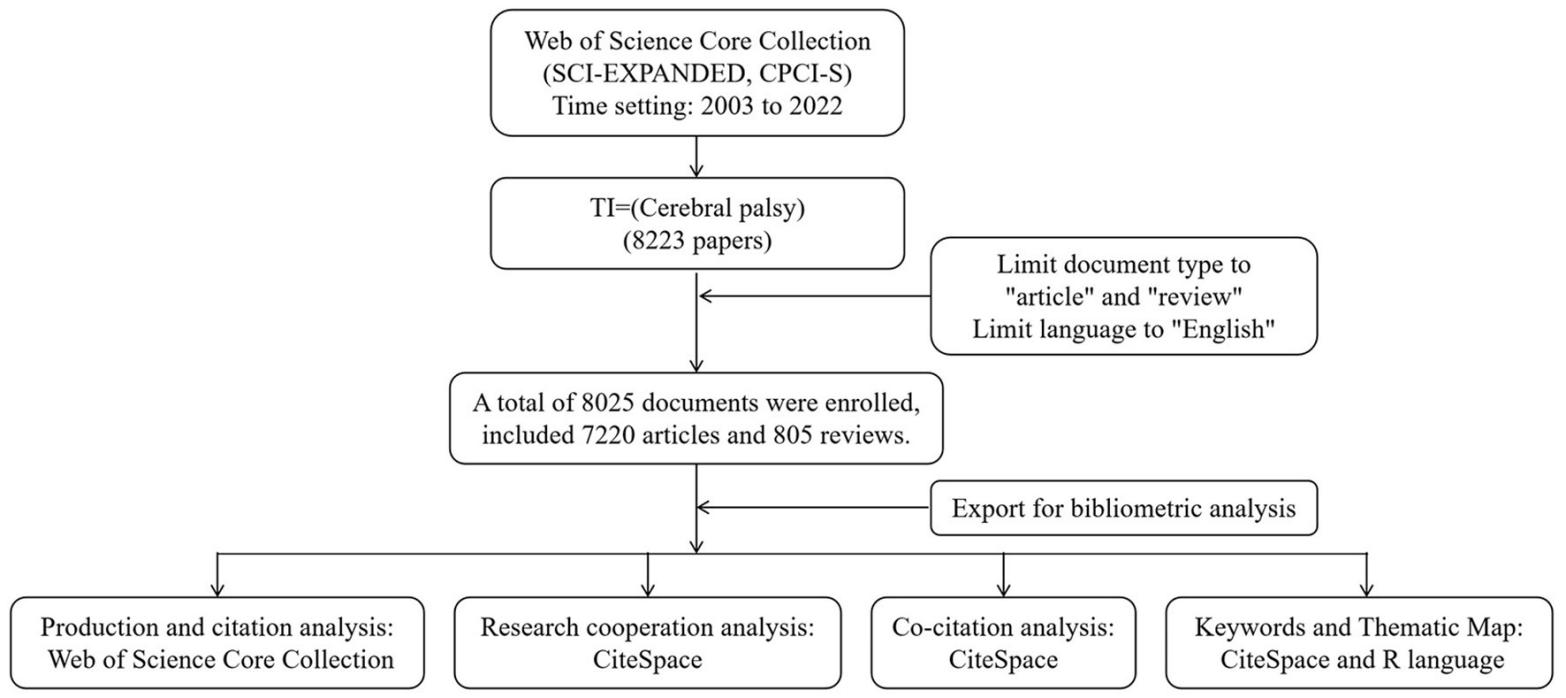
A flow chart of searching literature and analysis process.

The WOSCC can automatically analyze the literature related to a specific research topic, including countries, institutions, authors, and citation statuses. Accordingly, we searched for CP-related articles in the WOSCC (SCI-EXPANDED and CPCI-S) databases on 28 March 2023. Our search strategy was [TI = (“Cerebral palsy”)]. We targeted the literature from 2003 to 2022 and included articles and reviews among the document types, as follows. First, we conducted a WOSCC-based literature review to obtain general information and citations. In addition to the independent number of articles and citations, we used two comprehensive indicators, the H-index and G-index, to analyze the influence of authors and institutions. These two indicators relate to the number of papers and citations, respectively. The H-index indicates that, among all the articles by authors or institutions, there are at least H number of papers, and each has been cited at least H times. For example, if the H-index is 10, then there are at least 10 articles, each of which has been cited more than 10 times, and the larger the H-index, the greater the influence. The G-index categorizes the articles from high to low by citation, accumulates the number of citations by serial number, and squares the serial number. When the square of the serial number is equal to that of the cumulative number of citations, the serial number constitutes the G-index. If the square of the serial number is not exactly equal to but is smaller than the cumulative number of citations, then the serial number closest to the cumulative number of citations constitutes the G-index. Second, we used CiteSpace software (6.1.R3) to analyze the research collaborations using the occurrence of institutions, authors, and countries. Third, to better comprehend the scientific foundation of the CP-related research, we clarified the research base by co-citing references, authors, and journals. Finally, we used a keyword co-word analysis in CiteSpace and thematic maps in R to identify future research hotspots and trends. Figure 1 presents the flowchart of the literature search and analysis process.

Regarding the CiteSpace parameters, the slicing time was 2003–2022 with 1 year per slice. The node types included the authors, institutions, keywords, countries, references, cited authors, and cited journals; only one node was selected each time. In the map created using CiteSpace, the node thickness was proportional to the number of publications or citations. The outer color of the node (from blue to red) indicated the time, from early to recent (18). The outer purple ring of the node represented the centrality, that is, the number of nodes as the bridge for the shortest path between two other nodes, and the thicker the border, the stronger the intermediary centrality. An intermediary centrality value exceeding 0.1 indicated that the node played a vital role in the knowledge graph network. The line between the two nodes indicated a cooperative or co-citation relationship between them, and the thickness of the line represented the closeness of the two nodes. We also conducted a cluster network analysis of the co-cited references, co-cited authors, and keyword co-words. We measured the network modularity using the Q value of the cluster network. When the Q value exceeded 0.3, the network cluster structure was considered significant, and the larger the Q value, the better the network cluster. We assessed the homogeneity of the cluster network using the silhouette values. When the silhouette value exceeded 0.7, the cluster network had high reliability, and the closer the silhouette value was to 1, the higher the cluster network's homogeneity.

The R software integrates statistical analyses and graphical displays (19). The keyword thematic maps generated by Biblioshiny using the R language can be divided into four categories: the upper left corner is the motor themes (important and well-developed), which are the core themes with high maturity; the upper right corner is the isolated themes with high maturity niche themes (highly developed but unimportant in the current sector); the bottom left corner is the emerging or declining themes, new themes, or disappearing themes (marginal themes with little development, may have just occurred, or may soon disappear); and the bottom right corner is the basic themes and primary themes with low maturity, which may become research hotspots in the future.

## 3 Results

### 3.1 Publication outputs

**Figure 2:**
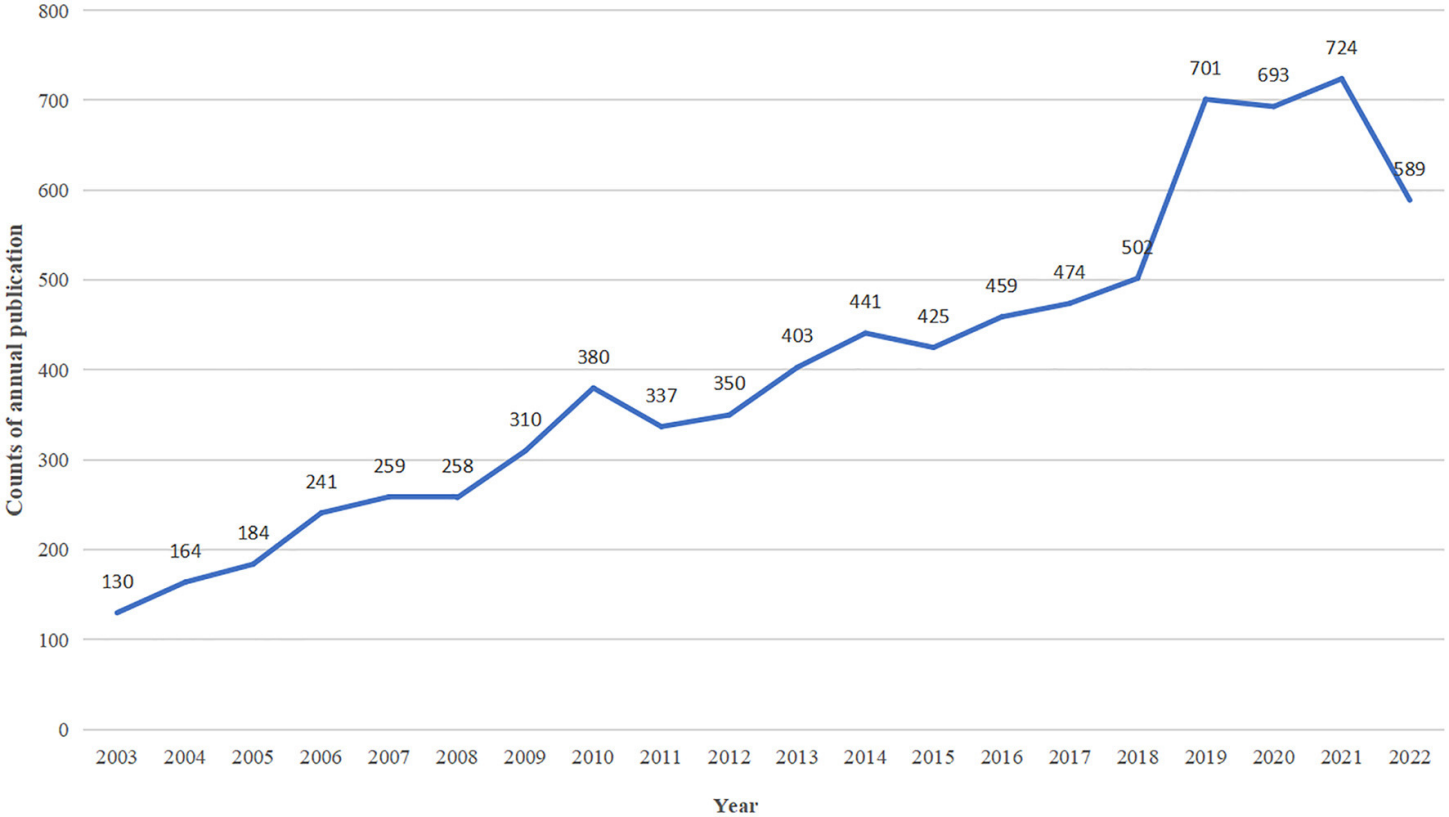
The annual number of publications from 2003 to 2022.

A total of 8,223 articles on CP were chosen after meeting the requirements. When the language was limited to English, 8,025 publications remained, including 7,220 articles and 805 reviews, with an average of 22.4 citations. Figure 2 illustrates the annual aggregate publications. The research period was divided into two stages: 2003–2018 and 2019–2022. Despite minor fluctuations during the initial stage, the overall growth rate increased by approximately 25 papers annually. The overall number of papers showed rapid growth in the first stage and then followed a stable development trend in the second stage. In 2003, there were a total of 130 articles on CP, which exceeded the 200, 400, and 700 articles in 2006, 2013, and 2019, respectively. In total, 4,023 articles received funds from the major funding sources; Table 1 presents the top 10 major funds. The top three major funding sources were the US Department of Health and Human Services, the National Institutes of Health, and the National Health and Medical Research Council of Australia.

**Table 1:** The top 10 funding sources.

**Ranking**	**Funding sources**	**Country**	**Frequency**
1	United States Department of Health and Human Services	USA	607
2	National Institutes of Health (NIH)	USA	570
3	National Health and Medical Research Council of Australia (NHMRC)	AUSTRALIA	292
4	Nih Eunice Kennedy Shriver National Institute of Child Health Human Development (NICHD)	USA	182
5	Canadian Institutes of Health Research (CIHR)	CANADA	133
6	NIH National Institute of Neurological Disorders Stroke (NINDS)	USA	106
7	National Natural Science Foundation of China (NSFC)	CHINA	94
8	Conselho Nacional De Desenvolvimento Cientifico E Tecnologico Cnpq	BRAZIL	80
9	Coordenacao De Aperfeicoamento De Pessoal De Nivel Superior Capes	BRAZIL	71
10	European Commission	European	64

### 3.2 Journals

From 2003 to 2022, a total of 1,008 journals published papers on CP. Table 2 presents the top 10 journals with the most published literature. These journals published approximately 33.9% of the total publications, while the top 20% accounted for 44.3% of the total. According to Bradford's law, the top 10 journals constitute the core journals of a research topic (20). Seven of the top 10 journals focused on child development or child rehabilitation, two focused on rehabilitation, and one focused on gait and posture. *Developmental Medicine and Child Neurology* was considered the most active journal, with 1,001 publications, which was approximately three times that of the second most active journal, *Disability and Rehabilitation*. The H-index of *Developmental Medicine and Child Neurology* was the highest (93) followed by *Archives of Physical Medicine and Rehabilitation* (43) and *Disability and Rehabilitation* (41). Table 2 presents the G-indices of the journals. Eight of the top 10 journals had an IF of >2.00 and two had an IF of >4.00; *Developmental Medicine and Child Neurology* had the highest IF (4.864). Despite the lack of journals with an IF of >5.00, two were classified using the Journal Citation Reports (JCR) Q1 region: *Developmental Medicine and Child Neurology* (pediatrics: Q1 zone) and *Archives of Physical Medicine and Rehabilitation* (rehabilitation: Q1 zone). On average, the CP-related publications in these two journals were cited more than 30 times. Regarding the number of articles and their influence (as reflected by their citations and JCR divisions, respectively), these two journals comprised the core journals for the CP-related research.

**Table 2:** The top 10 journals with the most published literature from 2003 to 2022.

**Ranking**	**Journal**	**Publications**	**No. of times cited**	**No. of times cited (per article)**	**Citation density**	**H-index**	**G-index**	**IF (2021)**	**JCR**
1	Developmental Medicine and Child Neurology	1,001	4,4882	44.84	3.8	93	160	4.864	CLINICAL NEUROLOGY Q2; PEDIATRICS Q1
2	Disability and Rehabilitation	321	6,521	20.31	1.9	41	65	2.439	REHABILITATION Q2; REHABILITATION Q2
3	Gait Posture	276	5,424	19.65	1.9	39	53	2.746	NEUROSCIENCES Q4; ORTHOPEDICS Q2; SPORT SCIENCES Q3
4	Journal of Pediatric Orthopedics	232	4,681	20.18	1.5	36	53	2.537	ORTHOPEDICS Q3; PEDIATRICS Q3
5	Developmental Neurorehabilitation	172	2,069	12.03	1.3	25	36	1.907	CLINICAL NEUROLOGY Q4; PEDIATRICS Q3; REHABILITATION Q3
6	Journal of Child Neurology	163	3,793	23.27	2.0	34	51	2.363	CLINICAL NEUROLOGY Q3; PEDIATRICS Q3
7	Archives of Physical Medicine And Rehabilitation	155	5,382	34.72	2.8	43	62	4.06	REHABILITATION Q1; SPORT SCIENCES Q2
8	Pediatric Physical Therapy	155	1,815	11.71	1.3	23	31	1.452	PEDIATRICS Q4; PEDIATRICS Q4
9	Physical Occupational Therapy in Pediatrics	133	1,696	12.75	1.5	25	32	2.297	PEDIATRICS Q3; REHABILITATION Q2;REHABILITATION Q2
10	Child Care Health and Development	114	2,453	21.52	1.9	29	44	2.943	PEDIATRICS Q2; PSYCHOLOGY, DEVELOPMENTAL Q3

### 3.3 Authors

The CP research from 2003 to 2022 revealed that 20,637 authors had written and published articles, while 23.2% of all authors had international co-authorship. Table 3 presents the top 10 authors with the highest number of articles. The top three authors were Boyd (143) from the University of Queensland, Miller (107) from Nemours/A.I. duPont Hospital for Children, and Gorter (90) from Network Childhood Disability Research. Regarding citations, Novak from the Cerebral Palsy Alliance ranked 10th in terms of publication numbers and performed best in terms of citations (58.5 citations per article). Boyd had the highest H-index, whereas Graham (from the Royal Children's Hospital of the University of Melbourne) and Novak had the highest G-indices.

**Table 3:** The top 10 active authors with the most publications from 2003 to 2022.

**Ranking**	**Authors**	**Institutions**	**Publications**	**No. of times cited**	**No. of times cited (per article)**	**Citation density**	**H-index**	**G-index**
1	Boyd RN	The University of Queensland	143	4,011	28.05	3.3	34	50
2	Miller F	Nemours Alfred I duPont Hosp Children	107	2,230	20.84	1.8	30	41
3	Gorter JW	Network Childhood Disabil Res	90	2,514	27.93	2.5	27	43
4	Desloovere K	Ku Leuven	88	2,293	26.06	2.9	26	40
5	Badawi N	The University of Sydney	86	2,837	32.99	5.1	27	51
6	Becher JG	Vrije Universiteit Amsterdam	83	2,944	35.47	3.0	32	42
7	Graham HK	Royal Children's Hospital Melbourne	75	3,766	50.21	4.0	32	59
8	Gordon AM	Columbia University	74	3,240	43.78	4.6	31	52
9	Dallmeijer AJ	Vrije Universiteit Amsterdam	71	1,853	26.1	2.4	27	36
10	Novak I	Cerebral Palsy Alliance	66	3,859	58.47	7.4	28	59

**Figure 3:**
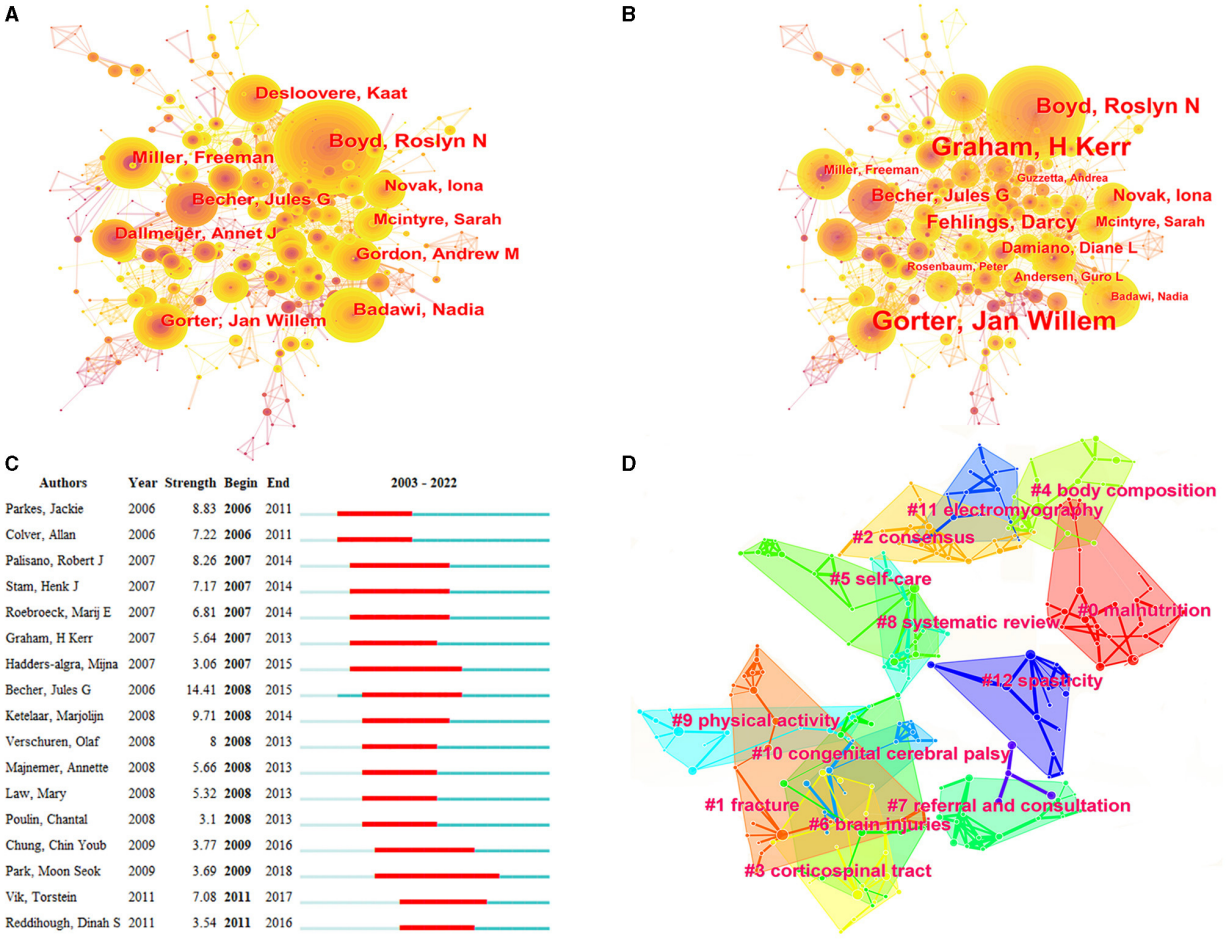
Co-occurrence map of authors. **(A)** Top 10 authors in citation counts. **(B)** Top 10 authors in centrality. **(C)** Top 17 strongest bursts authors. **(D)** A clustering map of the co-citation network of authors.

Figure 3A presents the maximal subnetwork for co-authorship, which was created using CiteSpace. When ranked by co-occurrence count, the top three results were Boyd (124), Badawi (73), and Miller (71). In terms of centrality ranking, the top three authors were Novak, Hambleton, and Ni (Figure 3B). The authors with the most articles also had substantial collaborations with others (Figure 3D). For example, Boyd conducted many clinical observations of children with CP, especially regarding upper limb motor function (21), and collaborated with Zi, Peter, and Davies. Figure 3C presents the authors with the strongest citation bursts. The top three authors were Boyd, Becher, and Peterson.

### 3.4 Institutions

**Figure 4:**
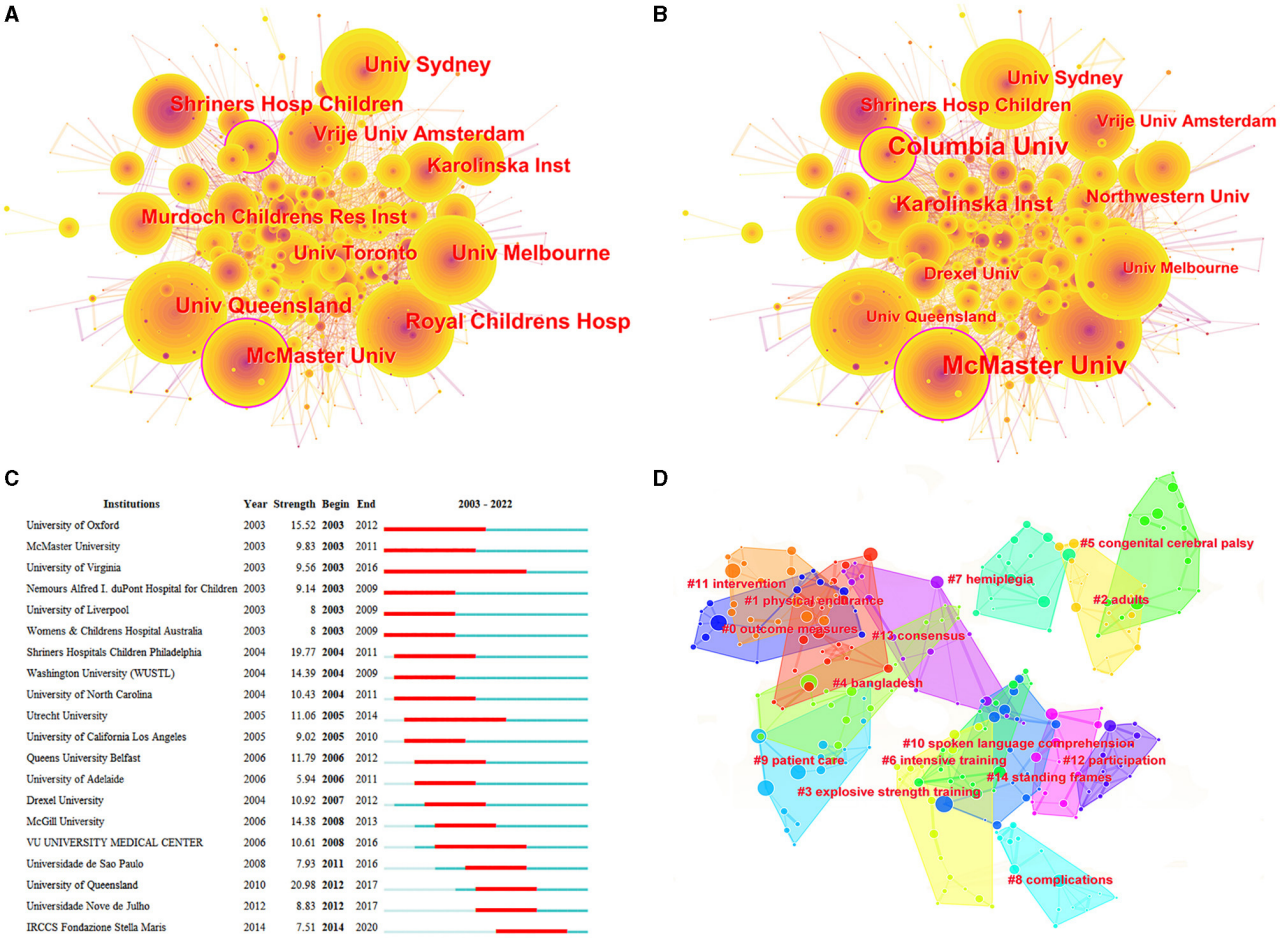
Co-occurrence map of institutions. **(A)** Top 10 institutions in citation counts. **(B)** Top 10 institutions in centrality. **(C)** Top 20 strongest bursts institutions. **(D)** A clustering map of the co-citation network of institutions.

Overall, 5,437 institutions contributed to the CP-related research from 2003 to 2022. Table 4 presents the top 10 institutions, while Figure 4 presents the top 10 institutions in terms of co-occurrence (Figure 4A) and centrality (Figure 4B). Regarding publications, Vrije Universiteit Amsterdam, the University of Queensland, and the University of Sydney were the top three institutions, while the gaps among the top seven institutions were very small, with a publication volume of approximately 200. The University of Melbourne and the Murdoch Children's Research Institute had the highest H-indices, while McMaster University ranked first in terms of citations, with an average of 50 citations per article. Vrije Universiteit Amsterdam, the University of Queensland, and the Royal Children's Hospital, Melbourne were the top three institutions in terms of co-occurrence, while the University of North Carolina System, the University of California System, and the National Institute of Neurological Disorders and Stroke (NINDS) were the most prominent institutions regarding centrality. In terms of articles produced, seven colleges were among the top 10 in terms of collaboration (Figure 4C). The top three institutions were Shriners Children's Hospital (2002–2009), the University of Oxford (2003–2012), and the University of Queensland (2012–2017). Figure 4D shows that each of these institutions had strong contact with other institutions. For example, the Royal Children's Hospital, Cerebral Palsy League, and Queensland HLTH collaborated with the University of Queensland. Meanwhile, the University of South Carolina System cooperated extensively with more than 10 institutions, including Boston University, Michigan State University, Harvard University, Baylor College of Medicine, Duke University, and Hartford University. Surprisingly, cooperation among the top institutions was relatively limited.

**Table 4:** The top 10 institutions with the most publications from 2003 to 2022.

**Ranking**	**Institutions**	**Countries**	**Publications**	**No. of times cited**	**No. of times cited (per article)**	**Citation density**	**H-index**
1	Vrije Universiteit Amsterdam	Netherlands	242	6,156	25.44	2.5	41
2	The University of Queensland	United Kingdom	239	6,507	27.23	3.1	43
3	The University of Sydney	Australia	225	6,390	28.4	3.7	43
4	Royal Children's Hospital Melbourne	Australia	223	8,184	36.7	3.5	49
5	The University of Melbourne	Australia	207	7,724	37.31	3.9	50
6	Murdoch Children's Research Institute	Australia	200	7,564	37.82	4.0	50
7	Mcmaster University	Canada	199	11,252	56.54	4.2	48
8	University of Toronto	Canada	174	4,673	26.86	3.1	36
9	Ku Leuven	Belgium	155	3,619	23.35	2.8	33
10	Karolinska Institute	Sweden	154	6,981	45.33	4.0	39

### 3.5 Countries/territories

**Figure 5:**
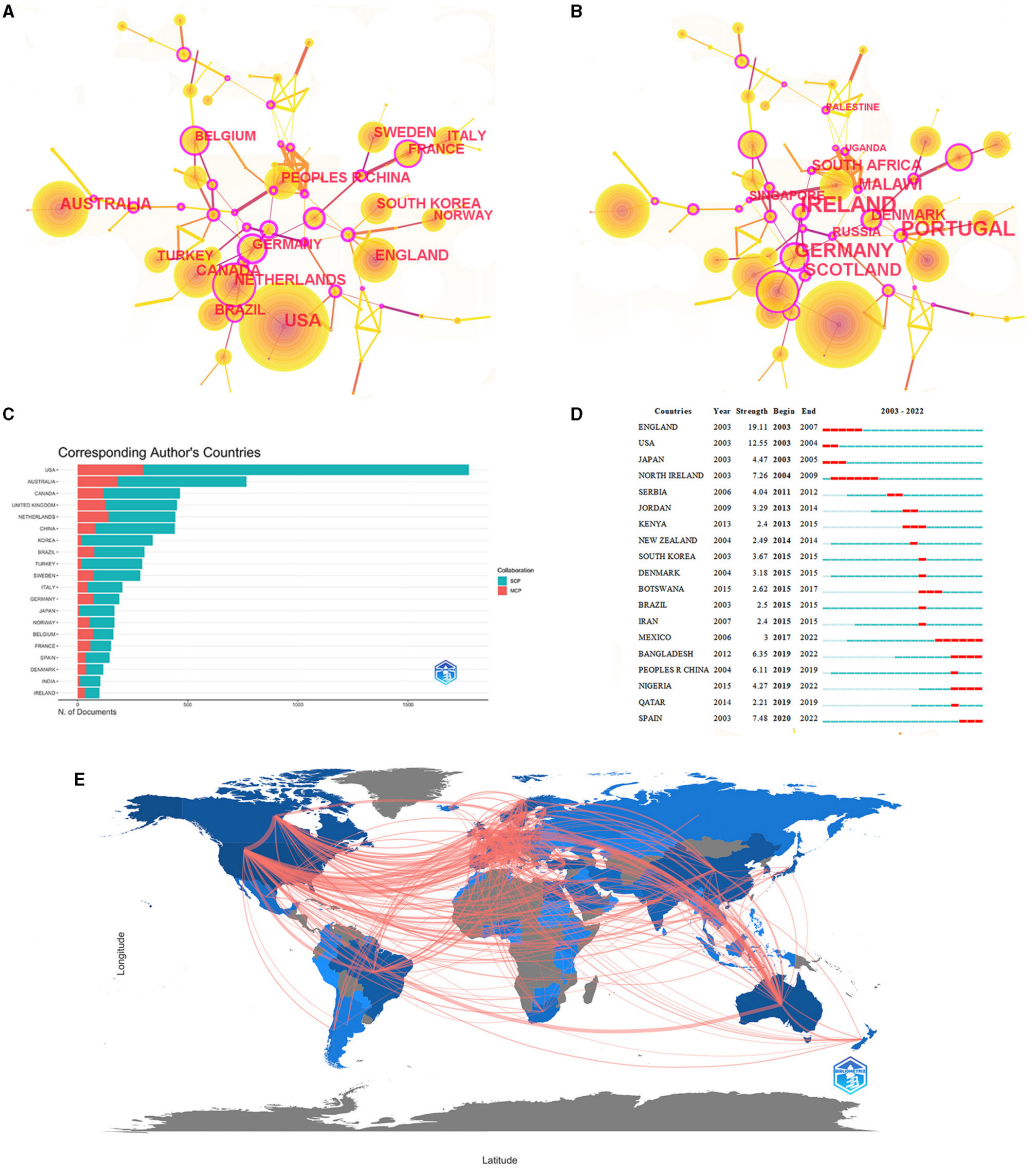
Co-occurrence analysis of countries and territories. **(A)** Top 10 countries in citation counts. **(B)** Top 10 countries in centrality. **(C)** The cooperation network of some countries. **(D)** Top 10 strongest bursts countries. **(E)** Country Collaboration Map.

Overall, 100 countries/territories had conducted CP research. Table 5 shows the top 10 countries/territories in terms of publications, citation density, and the H-index. The USA, Australia, and Canada ranked first, second, and third among the top three countries, with 2,197, 938, and 691 articles, respectively. Figure 5 shows the collaboration network map. When ranked by co-occurrence, the top three countries were the USA (2,105), Australia (869), and Canada (650) (Figure 5A). In terms of centrality, Israel, Portugal, and Germany were the three most prominent countries (Figure 5B). China ranked ninth worldwide, with 342 publications. Among the top 10 countries/territories, the USA had the highest H-index, while the top 6 countries had high H-indices exceeding 60. Regarding the average citation number, Sweden, Canada, and the United Kingdom (UK) performed better, with over 30 citations per article. China ranked last in terms of the H-index and citations.

**Table 5:** The top 10 countries/territories with the most publications from 2003 to 2022.

**Ranking**	**Countries/ territories**	**Publications**	**No. of times cited**	**No. of times cited (per article)**	**Citation density**	**H-index**
1	USA	2,197	62,670	28.53	2.6	97
2	Australia	938	27,352	29.16	3.1	76
3	Canada	691	23,279	33.69	3.1	67
4	England	625	20,824	33.32	3.0	72
5	Netherlands	570	16,471	28.9	2.7	61
6	Sweden	406	15,838	39.01	3.5	66
7	South Korea	367	5,571	15.18	1.5	37
8	Brazil	351	5,084	14.48	1.6	34
9	China	342	3,567	10.43	1.4	30
10	Turkey	325	3,755	11.55	1.3	30

Figures 5C, E show the cooperation among the countries/territories. Those with the highest numbers of international cooperation articles were the USA (multiple country publications [MCP]: 297), Australia (MCP: 183), the Netherlands (MCP: 140), the UK (MCP: 127), and Canada (MCP: 117). The top five countries with the most significant proportions of international cooperation articles were Belgium (MCP ratio: 45.1%), Switzerland (MCP ratio: 43.8%), France (MCP ratio: 38.4%), Germany (MCP ratio: 37.6%), and Iran (35.1%). The major collaborators with the USA were Canada (the frequency of cooperation: 176), Australia (the frequency of cooperation: 100), and the UK (the frequency of cooperation: 71), while China collaborated extensively with Sweden (the frequency of cooperation: 20) and the UK (the frequency of cooperation: 13). Portugal partnered with many countries, including Slovenia, Croatia, Hungary, Iceland, and Latvia. Additionally, the countries/territories with a greater number of published works, such as the USA and Australia, had more single-country publications; the top three countries were the UK, the USA, and Spain (Figure 5D). Regarding the co-occurrence, the USA, Australia, and Canada were the preeminent research powers in the CP field.

### 3.6 Co-citation analysis

The publications with the highest number of co-citations comprised the critical foundations of the CP-related research. Table 6 presents the top 10 co-citation articles. These were mostly guidelines and reviews on CP. None reported clinical trials but primarily focused on CP classifications, definitions, assessments, and therapy. The top co-cited article was by Rosenbaum et al., published in *Developmental Medicine and Child Neurology*, who re-examined the notion and categorization of CP by focusing on non-motor developmental issues and related musculoskeletal difficulties (22). The second most co-cited article was by Novak et al., published in *JAMA Pediatrics*, who evaluated the most dependable evidence for the diagnosis and early treatment of CP (23).

**Table 6:** The top 10 reference with most co-citation counts from 2003 to 2022.

**Ranking**	**Title**	**First author**	**Journal**	**IF**	**Frequency**	**Publication year**
1	A report: the definition and classification of cerebral palsy April 2006	Rosenbaum P	Developmental Medicine and Child Neurology	3.8	239	2007
2	Early, Accurate Diagnosis and Early Intervention in Cerebral Palsy Advances in Diagnosis and Treatment	Novak I	JAMA Pediatrics	26.1	202	2017
3	A systematic review of interventions for children with cerebral palsy: state of the evidence	Novak I	Developmental Medicine and Child Neurology	3.8	158	2013
4	Proposed definition and classification of cerebral palsy	Bax M	Developmental Medicine and Child Neurology	3.8	133	2005
5	Cerebral palsy	Graham HK	Nature Reviews Disease Primers	81.5	129	2016
6	An update on the prevalence of cerebral palsy: a systematic review and meta-analysis	Oskoui M	Developmental Medicine and Child Neurology	3.8	117	2013
7	Decreasing prevalence in cerebral palsy: a multi-site European population-based study, 1980 to 2003	Sellier E	Developmental Medicine and Child Neurology	3.8	111	2016
8	The Manual Ability Classification System (MACS) for children with cerebral palsy: scale development and evidence of validity and reliability	Eliasson AC	Developmental Medicine and Child Neurology	3.8	102	2006
9	Content validity of the expanded and revised Gross Motor Function Classification System	Palisano RJ	Developmental Medicine and Child Neurology	3.8	100	2008
10	State of the Evidence Traffic Lights 2019: Systematic Review of Interventions for Preventing and Treating Children with Cerebral Palsy	Novak I	Current Neurology and Neuroscience Reports	5.6	75	2020

Citation bursts refer to studies that are frequently cited in a given period and reflect the development dynamics of specific fields and influential literature. Recent high-citation bursts can reveal a discipline's research frontiers and hotspots, from which future development trends can be projected. In recent years, the most frequently cited article was “Early, Accurate Diagnosis and Early Intervention in Cerebral Palsy: Diagnosis and Treatment Advances (24).” To gain the most up-to-date understanding of early CP interventions, we thoroughly reviewed the literature on the best early diagnosis predictive tools for detecting CP risk before and after 5 months of age. Such tools should be mastered and fully applied by clinicians to adopt timely and early interventions to maximize their function and prevent secondary complications.

**Figure 6:**
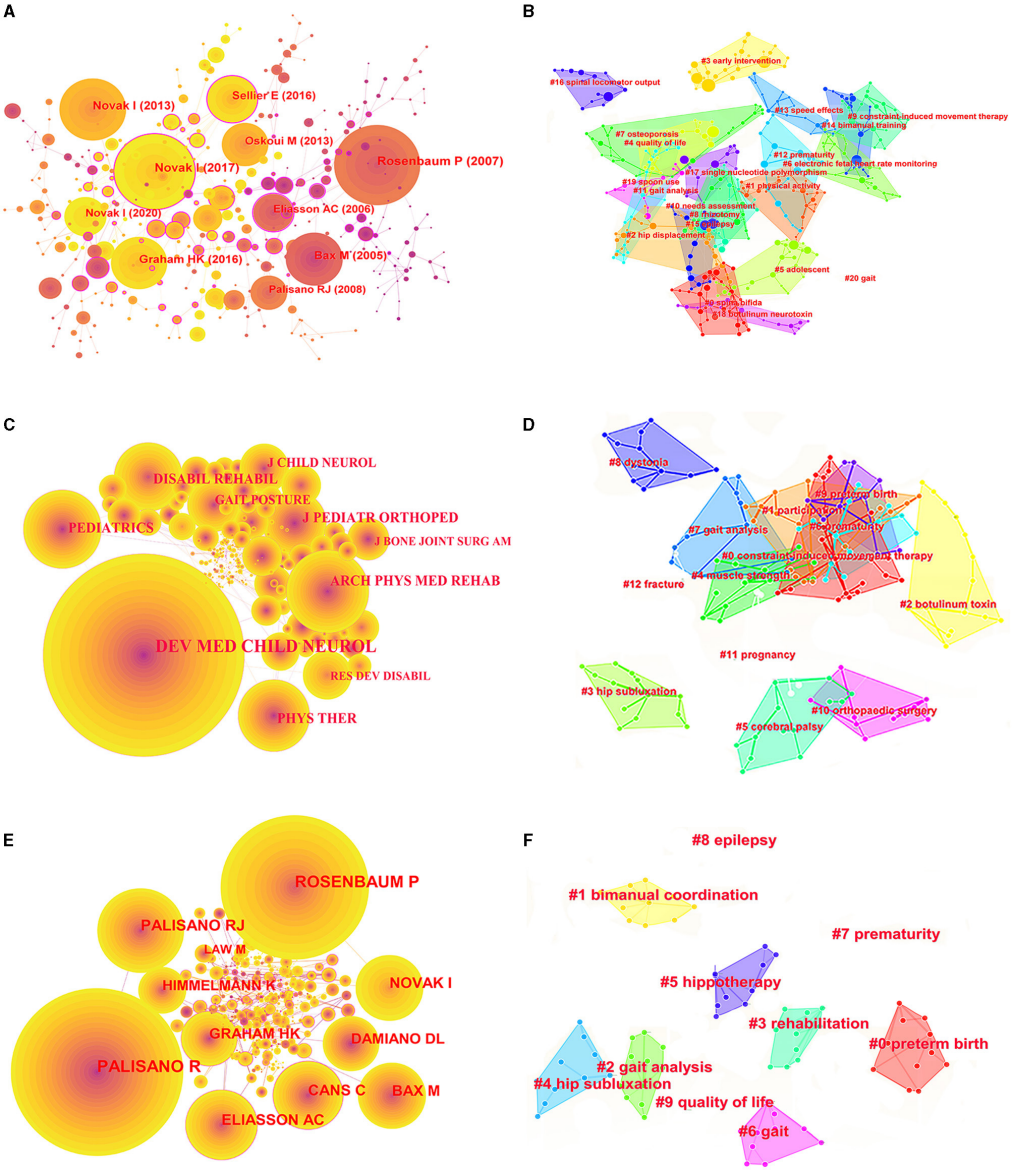
Co-citation analysis of references, journals, and authors. **(A)** Top 10 references in co-citation counts. **(B)** A clustering map of the co-citation network of references. **(C)** Top 10 journals in co-citation counts. **(D)** A clustering map of the co-citation network of journals. **(E)** Top 10 authors in co-citation counts. **(F)** A clustering map of the co-citation network of authors.

The cluster map of the co-cited articles consisted of 21 clusters, with a Q value of 0.8881. Figures 6A, B show the co-citation reference maximal subnetwork and cluster map, respectively. Based on the cluster label analysis, the research foundations from 2003 to 2022 comprised physical activity, magnetic resonance imaging (MRI), magnetoencephalography, periventricular white matter damage, gait, single nucleotide polymorphisms, and spinal locomotor output.

Figures 6C, D depict the most extensive subnetwork and cluster map of the co-cited journals, respectively. When ranked by the co-citation count, the top three journals were *Developmental Medicine and Child Neurology, Archives of Physical Medicine and Rehabilitation*, and *Pediatrics*. The top 10 publications also had an 80% overlap with the top 10 co-cited journals. The articles published in the top journals reflected the foundations of the CP-related research. Figures 5F, 6E show the maximal subnetwork and cluster map of the co-cited authors, respectively. Palisano (2,192 counts), Rosenbaum (1,831 counts), and Palisano (1,049 counts) were the top three authors with the highest co-citation counts. Consequently, these authors have established the groundwork for future CP research.

### 3.7 Keywords

**Figure 7:**
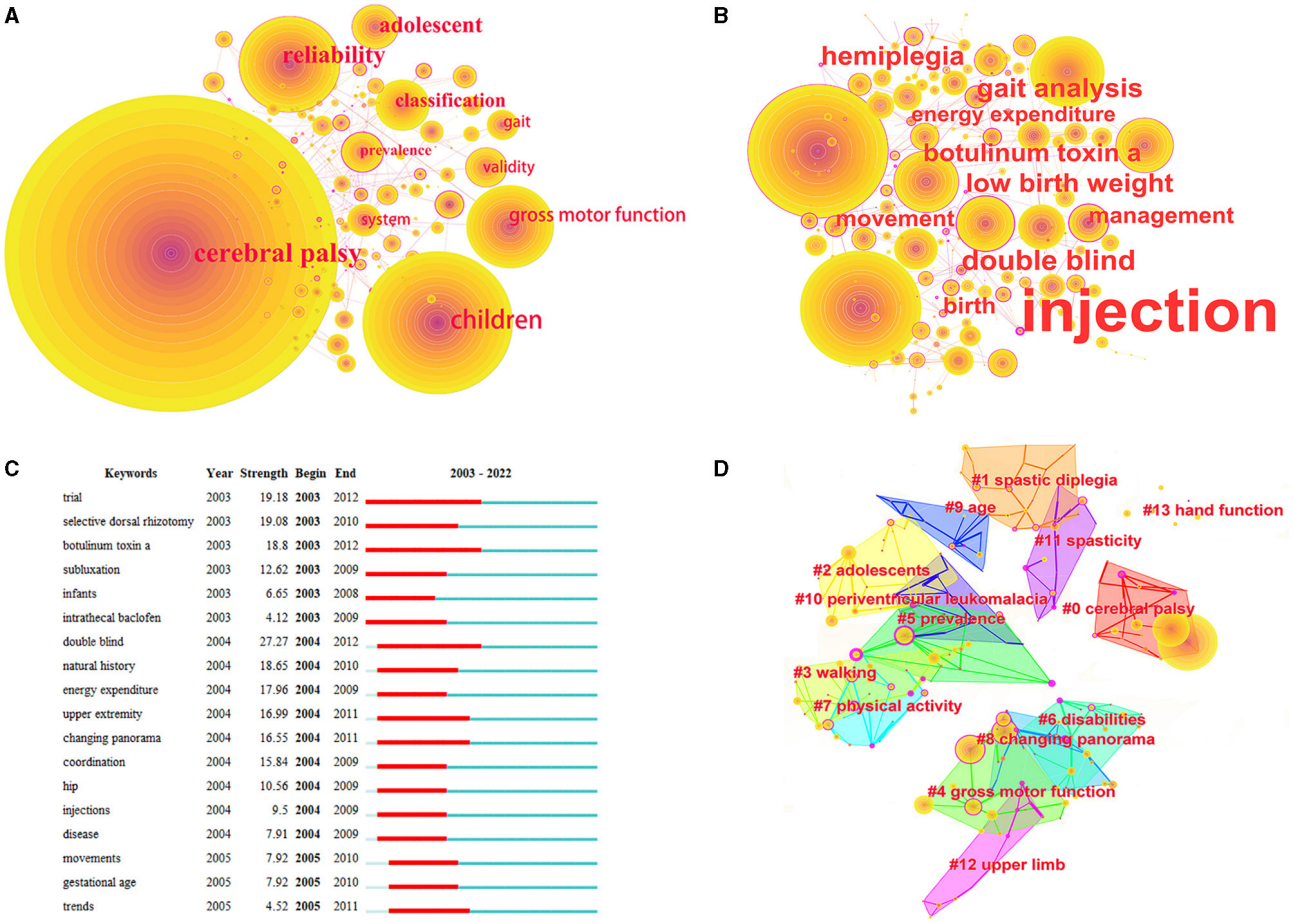
Co-occurrence map of keywords. **(A)** Top 10 keywords in citation counts. **(B)** Top 10 keywords in centrality. **(C)** Top 18 strongest bursts keywords. **(D)** A clustering map of the co-citation network of keywords.

The most common keywords were gait (553), rehabilitation (440), spasticity (325), botulinum toxin (174), therapy (148), upper extremity (141), quality of life (140), disability (115), pain (98), electromyography (97), kinematics (90), balance (88), participation (85), and walking (79). The most frequent keywords were classification, gait, quality of life, walking, spasticity, participation, outcome, efficacy, function, classification system, and risk factors (Figure 7A). The top three keywords in terms of centrality were efficiency, therapy, and energy expenditure (Figure 7B). The top-ranked keywords included double blind, randomized controlled trial, induced movement therapy, hand function, diagnosis, trial, selective dorsal rhizotomy, exercise, and botulinum toxin (Figure 7C). Regarding the keyword cluster analysis (Figure 7D), the keyword co-word network had 14 clusters and a Q value of 0.8497. In total, 13 clusters had a node number <10 and a silhouette exceeding 0.839.

**Figure 8:**
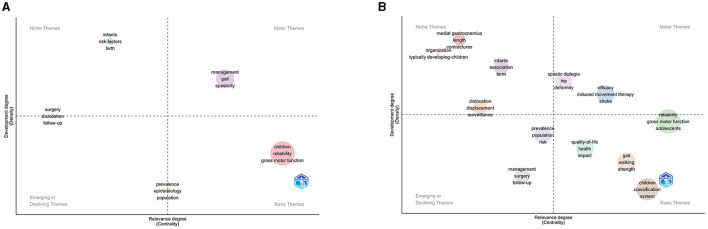
The keyword thematic map. **(A)** Keyword thematic map from 2003 to 2015. **(B)** Keyword thematic map from 2016 to 2022.

**Figure 9:**
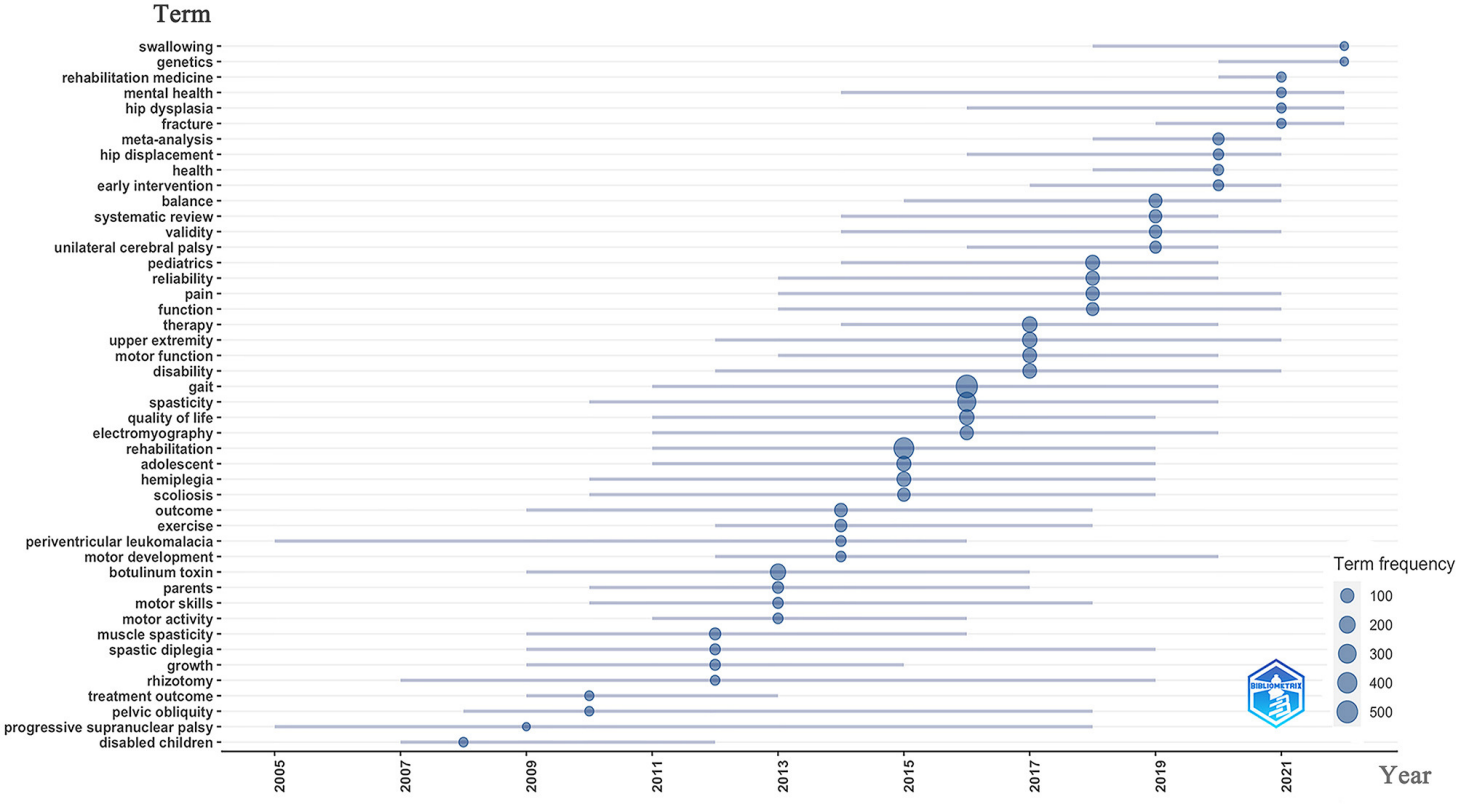
The change trends in the topic terms.

Figure 8 shows the keyword guide strategy coordinate map. We divided the thematic evolution into two periods, with 2016 as the boundary period. The third quadrant revealed management, surgery, follow-up, and prevalence, while the fourth quadrant revealed reliability, gross motor function, and gait. From 2016 to 2022, the themes indicating important and well-developed topics included spastic diplegia, deformities, efficacy, and induced movement therapy. The topic terms also changed from 2003 to 2022 (Figure 9). Those that appeared more frequently in recent years included swallowing, genetics, rehabilitation medicine, hip dysplasia, fracture, hip displacement, and pain, whereas earlier research topics included motor skills, botulinum toxin, exercises, and muscle spasticity.

## 4 Discussion

We performed a bibliometric analysis of the CP-related research on the WOSCC using CiteSpace and R software. We found that the number of publications steadily increased from 2003 to 2022. The most prolific and frequently co-cited journals were *Developmental Medicine and Child Neurology*. Boyd was the most productive and influential author, with 143 publications and 4,011 citations. The most fruitful institutions and countries were Vrije Universiteit Amsterdam and the USA, respectively. The core research forces were researchers and institutions from the USA, Australia, and Canada, with extensive collaboration in the CP-related research worldwide. Gait, botulinum toxin, spasticity, gross motor function, and therapy were the most commonly used keywords. Our findings offer valuable insights into the CP topic hotspots and future research directions.

### 4.1 Publication outputs and collaboration

Despite the extensive scientific research conducted between 2003 and 2022, CP remains a clinically challenging disease. We found that this field was developing, and research papers were gradually increasing, with results similar to those of the earlier bibliometric studies on CP (14). Over 600 articles were published from 2021 to 2022, suggesting great scope for further study. Medical research was affected by many factors, including the medical level, prevalence, economy, and technology. The research on CP significantly varied between countries/territories. Generally, mainly Western countries (such as the USA, Australia, Canada, the UK, and the Netherlands) had higher research outputs; the top 10 institutions and authors were also from these countries. Moreover, Western countries were also the major contributors to the pediatrics and rehabilitation research, which closely relates to CP (25, 26). Although there was little difference in the prevalence rate among various countries, considering the population base, the amount of CP research in populous countries, such as China, India, and the USA, might have been significantly higher than that of other countries. Based on the number and influence of CP research articles and funding sources, the USA was a major contributor. The volume and influence of the research from China and India were considerably smaller. The funding source data revealed that Australia and Canada also provided great support for CP research, and the impact (reflected in the number of articles and citations) showed that both countries were significant contributors.

China had a limited impact according to the citation count. Based on the epidemiological data, the number of CP cases in China was significant, thus further studies on CP should be conducted. However, the total number of studies in this field was far less than expected. China had a high number of articles in many domains; for example, there were 260 articles on childhood cancer published annually from 2007 to 2016, which was considerably larger than the research results for CP (27). The relative lack of scientific research on CP in China may be attributed to multiple factors. For example, pediatrics and rehabilitation, which have the highest correlations with CP, began relatively late in China and has had limited development. Consequently, there is a relative shortage of medical personnel in these two fields. China has taken measures to promote the development of these shortcomings, such as by establishing undergraduate majors in pediatrics. In the future, China may contribute more to the CP field.

One of the reasons for the remarkable research achievements in CP in Western countries may be due to the promotion of data informatization. Almost all the major contributors to the CP research have established their own databases. For example, in 2006, the USA created a CP-related database (NINDS) (28), while the American Academy of Cerebral Palsy and Developmental Medicine (AACPDM) collaborated to develop a CP-specific common data element (29). In 2009, Australia merged all seven of its CP registries into a common database. In 2000, Europe established a CP survey and registration network —the Surveillance of Cerebral Palsy in Europe (SCPE) (30)—in 8 countries and has 25 active centers in 20 European countries (31). In 2017, Switzerland created the Cerebral Palsy Registry (Swiss-CP-Reg) (32). Finally, the Dutch CP Register systematically collects real-world surveillance and treatment data from children with CP (33). The establishment of the above databases has not only promoted the development of CP research but has also provided a framework for collaborative research to promote further cooperation in the CP field. In the future, it is worth exploring the integration of different databases and resources to promote cooperation in this field.

We found differences in the number of studies and the research content between developed countries and low- and middle-income countries. Data from the Global Cerebral Palsy Register for Low- and Middle-Income Countries (GLM-CPR) from Bangladesh, Nepal, Indonesia, and Ghana indicated that low- and middle-income countries had a high proportion of severe CP. The GLM-CPR has called for urgent action to identify prevention opportunities and promote early diagnosis and intervention in children with CP in low- and middle-income countries (34). The CP research in Brazil, India, and Africa has focused mainly on the body's functional and structural aspects, while activities and participation, environmental factors, such as resources and policies, and personal factors have not been fully explored; these are modifiable factors that can be addressed through interventions (35–37). Overall, these treatment-related studies have only focused on impairment rather than function and may have limited relevance for clinical decision-making. Therefore, the research in low- and middle-income countries should expand their focus beyond the body's functionality and structure to include participation and contextual factors. The implementation of evidence-based interventions (e.g., family programs, environmental enrichment, and task-specific training) does not require expensive equipment and is both affordable and feasible (38).

In an increasingly interconnected world, researchers should make full use of available resources, facilitate collaboration, and maximize efficiency. There has been extensive and close collaboration among the CP researchers worldwide, suggesting that the CP-related research has reached a certain level of maturity. While some have had limited publications, they have had many collaborations. For example, Galli (15) has extensively collaborated with Cimlin (4), Albertini (3), Brunner (13), and Oliveira (18), thereby promoting global collaboration. However, there has been relatively limited cooperation between top institutions (such as Vrije Universiteit Amsterdam and the University of Queensland) and top authors (e.g., Boyd and Miller). The previous research has revealed an inverted U-shaped relationship between research topic overlap and the likelihood that researchers will collaborate with each other, with the initial likelihood of collaboration increasing with topic similarity. After a “sweet spot” of similarity is reached, collaborative relationships decline rapidly because excessive overlap in research topics intensifies the competition and limits the potential for further collaboration (39). Generally, the top research forces have developed into a full-fledged stage. They have gradually formed their own specific and clear research directions during the development process and have enough strength to complete the research independently. Accordingly, many developing institutions or authors want to work with these top research forces (such as the University of Queensland and the University of Carolina) to gradually form a collaborative network centered on these top institutions.

### 4.2 Keyword co-word analysis

A keyword-based analysis can indicate a theme's evolutionary trends and research hotspots in a specific field of study. We found that the most frequently used terms in the CP-related research were classification, gait, quality of life, walking, spasticity, participation, outcome, and efficacy. The main research frontiers and hotspots are summarized in the following.

#### 4.2.1 Genetic factors have received much attention as one of the causes of CP

Increasing evidence has supported the role of genetic variations in the CP etiology. Exome sequencing has shown that the overall diagnosis rate of CP is 23–31.1% (40–42), and the detection rate in children (32.7–34.8%) is higher than that in adults (10.5–26.9%) (40, 43). The diagnostic yield of the chromosomal microarray is 5% (95% CI: 2–12) (42). In 2017, the International Cerebral Palsy Genomics Consortium (ICPGC) developed a platform to integrate genotypic, phenotypic, and imaging data with the availability of biobank samples, so as to make full use of such resources by sharing them with other databases (44). The precise diagnosis of metabolic or genetic causes is important for determining treatment possibilities, accurate prognosis, and genetic counseling, indicating that patients with CP should undergo genomic testing as part of their diagnostic program.

#### 4.2.2 Research related to the treatment of functional impairment

Motor dysfunction is the core symptom of CP; however, other functional impairments (e.g., sensory, perception, cognition, and communication), behavioral disorders (e.g., epilepsy), and secondary musculoskeletal diseases are common. The in-depth exploration of motor function recovery and treatment strategies for CP have always been popular research topics; of which, gait is the most important, as demonstrated by the third-ranked journal, *Gait and Posture*. Lerner et al.'s (45) published study in *Science Translational Medicine* (IF: 19.343) reveals that a wearable exoskeleton is a longer-lasting remedy for improving crouch gait and the quality of life than surgery, toxin injections, physical therapy, and robotic gait trainers. A meta-analysis also found that robotic exoskeletons could improve mobility in children with CP, thereby increasing community participation and improving their quality of life (46). Therefore, randomized controlled studies with larger sample sizes or multiple centers should be conducted in the future. However, despite numerous efforts undertaken to identify more effective controllers for exoskeleton-based gait rehabilitation, there is scant evidence regarding the effectiveness of different control strategies on clinical outcomes (47). Many issues remain unresolved, such as high heterogeneity, due to the lack of standardized experimental protocols. Analyzing the relationships between the control parameters and key biomechanical indicators and achieving standardized comparisons between different control strategies may better guide the future technological development of exoskeleton robot-assisted walking rehabilitation. Recent clinical trials have also revealed that treatment methods developed in neurorehabilitation have been widely studied in CP research, such as transcranial magnetic stimulation (48), virtual reality technology (48), whole-body vibration (49), wearable exoskeletons, and stem cell transplantation (45, 50–52).

Mesenchymal stem cell transplantation is a relatively new research hotspot and frontier in the medical profession and life sciences, with a remarkable effect on central nervous system illnesses and nervous dysfunction induced by major system injuries. Studies have revealed that the use of umbilical cord mesenchymal stem cells is a safe, practical, and successful way to enhance gross motor function in children with CP (52). Intranasal administration of neurogenic stem cells has been well tolerated and may be beneficial for patients with CP (53). One study has demonstrated stem cell therapy as an anti-inflammatory and anti-apoptotic for patients with CP (54), suggesting that it may be a promising adjunct to traditional CP therapies. However, the lack of effective models has hindered more in-depth research (55). Although stem cell therapy is not currently widely used in children with CP, the parents of these children remain optimistic about the potential improvement of stem cell therapy, given the limited treatment options available for CP (56). Accordingly, stem cell treatments are worthy of exploration and may lead to breakthroughs in the future.

#### 4.2.3 Complications and management of CP

Common challenges in the complications and management of CP include spasticity and dystonia, pain management, hip monitoring, sleep and feeding, swallowing, and nutrition (57). Common musculoskeletal and joint complications include ankle and foot deformities, hip displacement, a windswept posture, scoliosis, pain, and pelvic obliquity. Although equinus foot is the most common deformity in patients with CP, there is no clear clinical definition. However, Horsch (58) proposed a clinical cutoff of a ≤5° dorsiflexion as the recommended value for defining functionally relevant equinus foot in patients with CP. Casting, orthoses, and physical therapies, such as shock waves, botulinum toxin, and surgery, are common treatments for the equinus foot in patients with CP (59–61). Horsch evaluated gait outcomes and strength following different surgical procedures of the equinus foot in two groups of patients with CP and found that the Strayer procedure and Achilles tendon lengthening showed higher postoperative dorsiflexion. Moreover, an 8.2% loss in calf muscle strength was observed in the Strayer group, while in the Baumann procedure, no loss of strength was observed, and maximum power improved postoperatively (62). The hip is the second most commonly affected joint in patients with CP, thus hip surveillance programs are recommended to prevent, reduce, and identify hip dislocation (63, 64). Such programs for children with CP have been conducted in Europe, Oceania, and Canada. The age, the gross motor function classification system, and the migration percentage have been identified as the key factors in hip surveillance programs (65).

As the survival rate of children with CP increases, the survival rate of adults with CP will also increase. Adults with CP may experience many other health problems, such as more chronic health conditions, worsening physical activity, increased risk of musculoskeletal complications, progressive changes in swallowing ability, difficulty participating in social activities, and lower health-related quality of life (66). Moreover, the overall average pain prevalence in adults with CP is 70% (95%: CI: 62–78) (25). Schmidt et al. (67) revealed that bone/joint complications were more strongly associated with lower limb pain in patients with CP followed by reduced mobility. Lifestyle interventions carried out for 6 months can improve fatigue, physical pain, mental health, and social support in adolescents and young adults with CP, while physical behavior and health have significant mediating effects on fatigue, body pain, and psychological wellbeing (68).

#### 4.2.4 The outcomes of CP

A study that focused on CP in children and adolescents used the International Classification of Functioning, Disability, and Health (ICF) framework to identify the current research focus and gaps in CP and found that over 67.9% of the literature had investigated activities (67.9%) followed by body functions and structures (42.9%), participation (14.2%), and environmental factors (3.6%). An analysis of the outcome indicators in 274 of the adult CP-related studies suggested that 4,409 meaningful concepts were associated with the ICF, of which “activity and participation” were the most common followed by “physical function”; meanwhile, the most common category was “pain” (b280, 37.6%) followed by “walking” (d450, 33.3%) and “paid work” (d850, 27.5%) (69). One study found that many adults with CP were ambulatory, had few manual difficulties, and might experience fatigue and pain; on average, 40% were employed and 30% lived independently (70). Overall, the uniformity of the assessment and reporting of the outcome measures of concern for researchers, patients, and caregivers should be improved.

## 5 Conclusion

The CP research field is small but emerging and has grown in popularity over the past two decades. The significance of research on CP has been highlighted in *Developmental Medicine, Child Neurology, Disability and Rehabilitation*, and *Gait and Posture*. The most well-known author, institution, and country are Boyd, Vrije Universiteit Amsterdam, and the USA, respectively. Researchers and institutions in the USA, Australia, and Canada have substantially contributed to the CP topic. Global research collaborations are extensive but may require additional collaboration between top institutions, authors, and countries. Meanwhile, China and India should strengthen their research on CP. The CP-related research in countries/territories with limited resources should expand its focus beyond the body's functional and structural aspects, including participation and contextual factors, and implement evidence-based interventions that can be conducted locally. Genetic factors, emerging detection and treatment methods, complications, and clinical outcomes are popular topics in the CP field.

## 6 Limitations

This study has the following limitations. The first limitation is that although our bibliometric study summarizes the research achievements in the CP field from 2003 to 2022 through a quantitative analysis, the value of more recently published studies may have been overlooked. The second limitation is that because CP involves many disciplines, we were unable to cover all aspects, and some are very important, such as imaging studies (e.g., resting-state magnetoencephalography and resting-state functional magnetic resonance imaging) (71) and health policy services. The third limitation is in terms of our method; we only analyzed the literature recorded in the WOSCC. To the best of our knowledge, the current databases used in bibliometrics are the Web of Science, Scopus, and PubMed. In terms of the number of articles, PubMed and Scopus can provide larger samples than the WOSCC; however, PubMed cannot provide citation-related data. The WOSCC can limit papers to core journals and provide more information about citation data, impact factors, and JCR. Considering that not all three databases can provide the same information, merging publications from different databases may result in the loss of some information; therefore, we only chose to use the WOSCC. The fourth limitation is that when analyzing the citation count, we did not consider bias caused by self-citation. Moreover, there may have been a small number of omissions in the literature in 2022, as the WOSCC's literature record may lag behind that of the publications. Nevertheless, our results provide a general overview of the research trends in CP.

## Data availability statement

The original contributions presented in the study are included in the article/supplementary material, further inquiries can be directed to the corresponding author.

## Author contributions

YH: Conceptualization, Formal analysis, Writing – original draft, Writing – review & editing. YZ: Software, Visualization, Writing – original draft, Data curation. YY: Software, Writing – original draft, Visualization. WF: Software, Writing – original draft. MH: Methodology, Software, Validation, Writing – review & editing. DL: Data curation, Software, Validation, Writing – review & editing. ZX: Data curation, Software, Validation, Writing – review & editing. FX: Funding acquisition, Supervision, Writing – review & editing. JW: Funding acquisition, Writing – review & editing.
